# Successful Surgical Management of a Ruptured Intracranial Aneurysm in a Nine-Month-Old Male: A Rare Pediatric Case

**DOI:** 10.7759/cureus.63684

**Published:** 2024-07-02

**Authors:** Anirudh Kommareddy, Ashish Varma, Keta Vagha, Chaitanya Kumar Javvaji, Jayant D Vagha, Sai Bhavani Manchineni, Naramreddy Sudheesh Reddy

**Affiliations:** 1 Pediatrics, Jawaharlal Nehru Medical College, Datta Meghe Institute of Higher Education and Research, Wardha, IND

**Keywords:** aneurysm, pediatric stroke, pediatrics neurosurgery, intracranial bleed, mycotic aneurysms

## Abstract

Intracranial aneurysms in pediatric populations are rare, with a distinct clinical profile compared to adult cases. This case report describes the clinical presentation, diagnosis, and treatment of a nine-month-old male with an intracranial aneurysm. The child presented with convulsions, a depressed sensorium, and subsequent neurological deficits. Initial imaging revealed subarachnoid hemorrhage, and further angiographic studies identified an aneurysm rupture from the parietal branch of the right middle cerebral artery. The patient underwent successful neurosurgical intervention, including right craniotomy and aneurysm clipping. Post-operative recovery was marked by gradual neurological improvement and the absence of further seizures. This case underscores the importance of prompt diagnostic imaging and surgical management in pediatric intracranial aneurysms, contributing to favorable outcomes despite the rarity of the condition in this age group.

## Introduction

An unusual dilatation of a focal artery is called an aneurysm. Secondary infections can arise from pre-existing aneurysms, but bacteremia or septic embolization, as in the case of mycotic aneurysms, can also cause aneurysmal degeneration of the arterial wall. Thin-walled protrusions in the intracranial arteries, called cerebral aneurysms, have the potential to burst and result in a subarachnoid hemorrhage (SAH), which is frequently a catastrophic occurrence with a high death and morbidity rate. Unruptured cerebral aneurysms can also be clinically identified by their mass effect on other neurologic structures, or they can be unintentionally found during a patient's neuroimaging study performed for another purpose. The likelihood of rupture and SAH in the future for unruptured aneurysms varies depending on their size and location. Most patients with unruptured intracranial aneurysms (IAs) are asymptomatic. However, they can still present with symptoms such as visual acuity loss due to third cranial nerve compression, pyramidal tract dysfunction, facial pain, and cranial neuropathies. Sudden severe headache is a common indication where a CT scan was advised, leading to the detection of the aneurysm [1]. Ruptured IA can present with headache, convulsion, altered sensorium, loss of consciousness, vomiting, and neck pain. The risk factors for IA include hypertension, estrogen deficiency, and genetic factors leading to hereditary syndromes (Ehlers-Danlos syndrome, polycystic kidney disease, familial aldosteronism type I, moyamoya syndrome) associated with IA [2,3].

IAs are infrequent in children and adolescents. Pediatric aneurysms are generally accepted to be a separate condition from adult aneurysms despite the sparse and sometimes contradictory literature on cerebral aneurysms in people under the age of 18 [4]. Compared to adults, children have a far lower prevalence of aneurysms, and they are extremely uncommon during the first year of life. With a male-to-female ratio of 1.8:1, aneurysms in children affect boys more often than females [5]. Prior to the advent of angiography, the information regarding SAH and its etiologies relied heavily on autopsy findings. Determining the cause of surviving patients was challenging. The introduction of angiography revolutionized the field by enabling the diagnosis of SAH in living patients [6]. IAs are infrequent in infants, representing an estimated 0.5-4.5% of all aneurysms [7]. Presently, two primary modalities of treatment exist: endovascular and microsurgical approaches. Despite extensive research, conclusive evidence of superiority between these two methods has yet to be established [8].

## Case presentation

**Figure 1:**
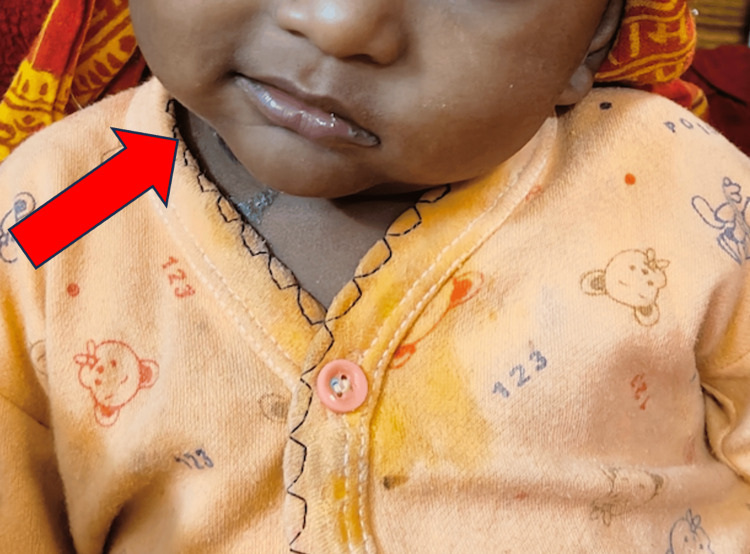
Physical examination revealing facial deviation towards the right side (red arrow)

A nine-month-old male child presented with a history of convulsions for two days and a depressed sensorium for one day. The mother reported that the child was previously well until he suddenly began vomiting after waking up from sleep, followed by a convulsion characterized by tightening of all four limbs, eye-rolling, and a staring look lasting four minutes. Post-ictal drowsiness was present. No history of fever, recent dental procedures, or genitourinary infections was reported. The child was initially evaluated at a local hospital, where a computed tomography (CT) scan revealed SAH, leading to referral to a tertiary care center. Upon admission to the pediatric intensive care unit, a detailed history was taken, followed by an examination. On examination, heart rate was 110 beats per minute, respiratory rate was 28 cycles per minute, and blood pressure was 98/62 mm of Hg. All peripheral pulses are well felt. On examination, the child was observed to have decreased movements in the left upper and lower extremities. The child had a deviation of mouth towards the right with drooling of saliva (Figure 1).

**Figure 2:**
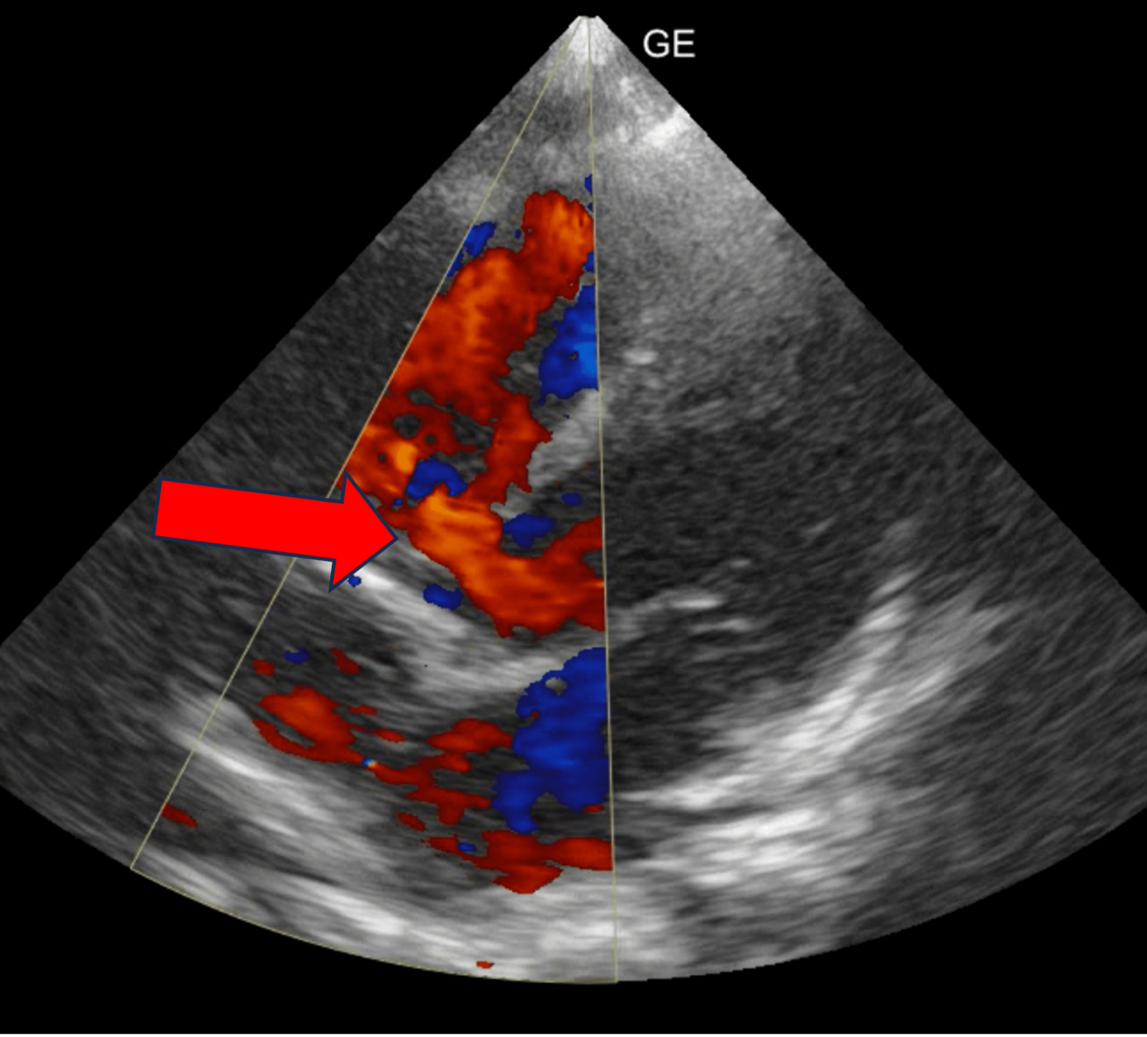
A 2D echocardiography revealed ventricular septal defect (red arrow).

The child was observed to be depressed activity also. On auscultation, a pan systolic murmur was heard. Respiratory and abdominal system examinations were normal. Investigations revealed a low hemoglobin level of 8.9 g/dL (reference range: 13-15 g/dL), elevated white blood cell count of 15,400 cells/cumm (reference range: 4,000-11,000/cumm), and thrombocytosis. Other investigations were normal. Lumbar puncture and cerebrospinal fluid analysis were unremarkable, and blood cultures showed no growth of any organism. The child was started on treatment with intravenous fluids, meropenem (40 mg/kg/day), vancomycin (60 mg/kg/day), Levera (40 mg/kg/day), and calcium gluconate. A 2D echocardiography revealed ventricular septal defect (Figure 2).

**Figure 3:**
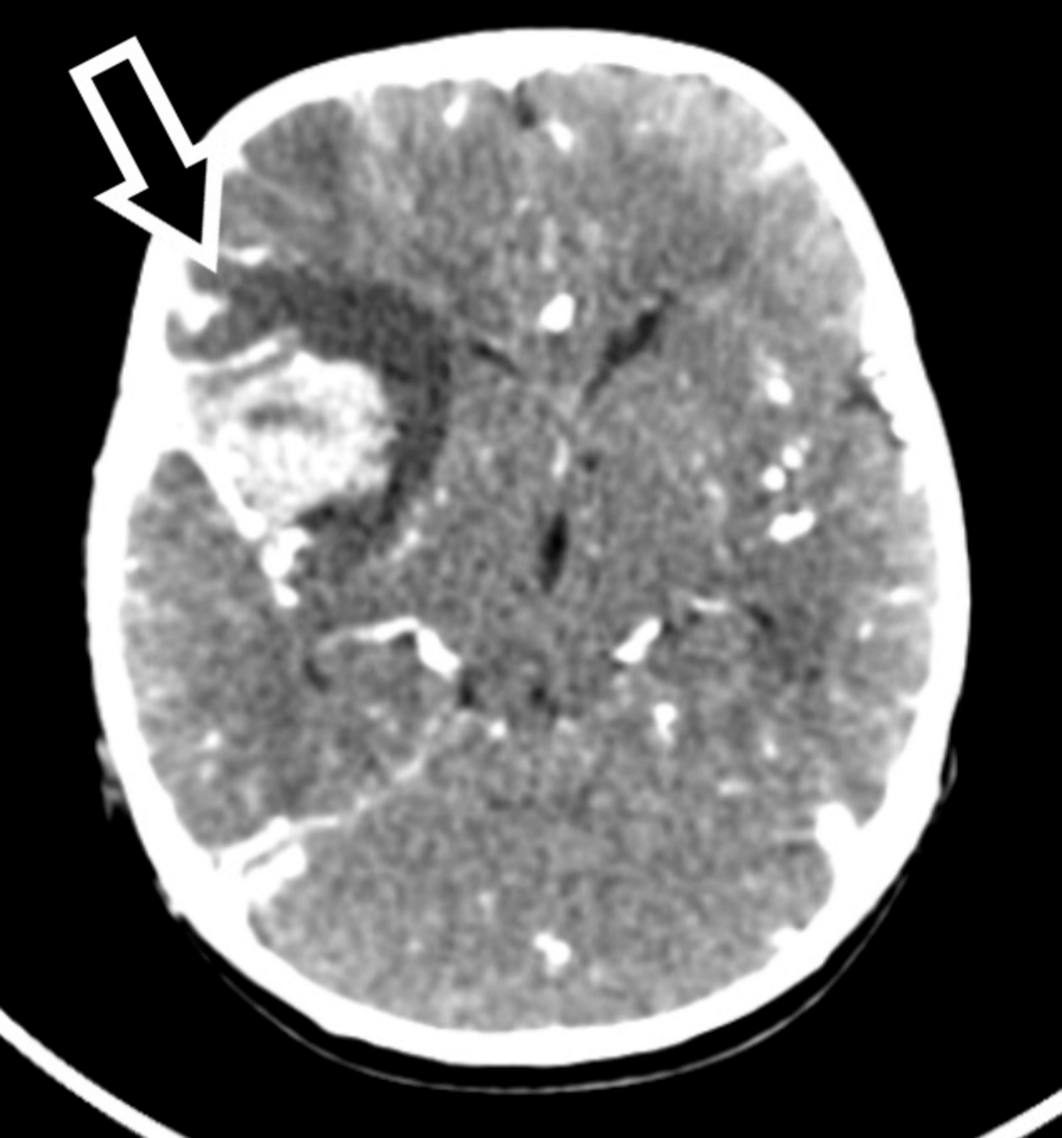
Brain computed tomography angiogram (axial section) showing intraparenchymal hemorrhage in the right frontotemporal lobe (right MCA territory) with adjacent subarachnoid component (black arrow) and edema causing a midline shift to the right side. MCA, middle cerebral artery

Brain CT angiogram showed intraparenchymal hemorrhage in the right frontotemporal lobe (right middle cerebral artery [MCA] territory) with adjacent subarachnoid component and edema causing a midline shift to the right side (Figure 3).

**Figure 4:**
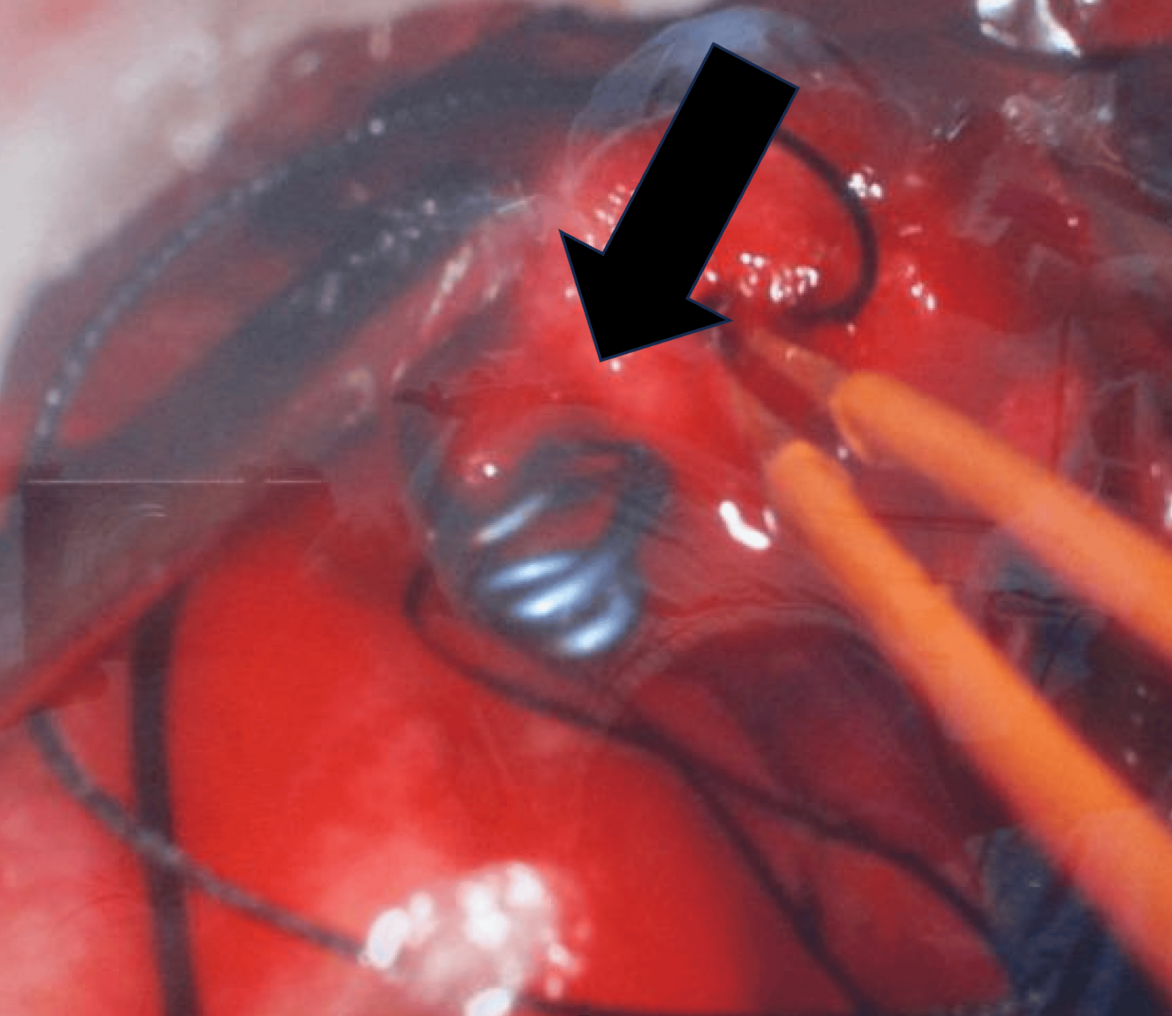
Intraoperative image showing clipping of the aneurysm (black arrow)

Neurosurgery performed a right craniotomy for clipping the aneurysm and evacuating the intracranial bleed (Figure 4).

**Figure 5:**
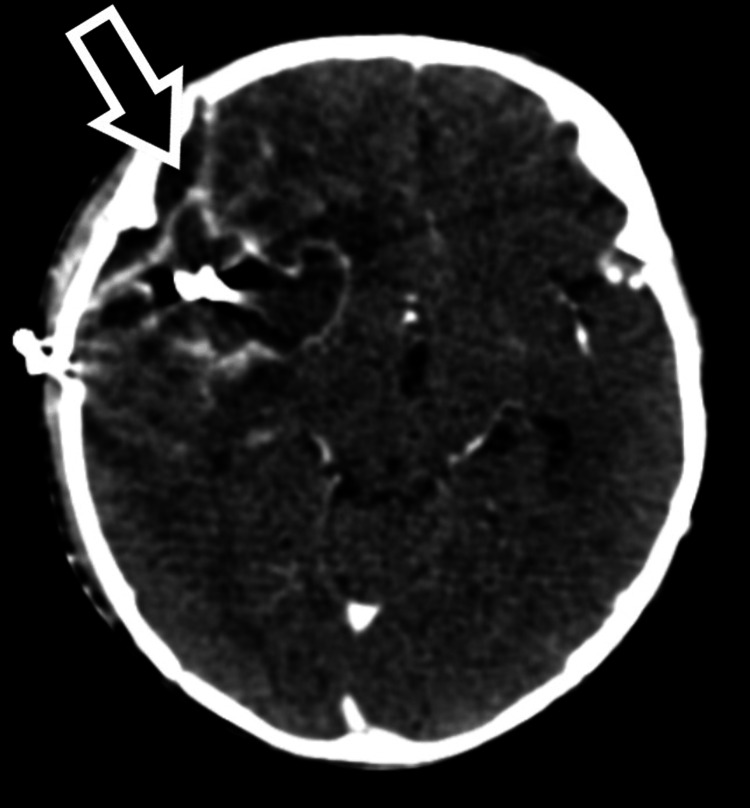
Post-operative computed tomography of the brain (plain axial section) showing resolving intraparenchymal hemorrhage with reduced midline shift and cerebral edema in the right frontotemporal lobe with metallic surgical clip in situ (black arrow), suggesting right MCA aneurysm clipping. MCA, middle cerebral artery

Post-operatively, the child remained stable and received transfusions to compensate for intraoperative blood loss. Post-operative brain CT showed resolving intraparenchymal hemorrhage with reduced midline shift and cerebral edema in the right frontotemporal lobe with metallic surgical clip in situ, suggesting right MCA aneurysm clipping (Figure 5).

The child's condition improved, with decreased weakness in the left upper and lower limbs and adequate limb movements. There were no further convulsions or vomiting episodes. Intravenous antibiotics were continued until day 9, followed by oral cefpodoxime (10 mg/kg/day) and Levera (40 mg/kg/day). The patient was discharged with instructions for a follow-up appointment after seven days.

## Discussion

Pediatric IAs are notably less common than in adults, particularly infants, and their etiology remains poorly understood. Contrary to adult cases, pediatric IAs often exhibit fusiform morphology rather than saccular and tend to be more prominent and asymmetrical [4]. Lasjaunias et al. proposed that pediatric IAs are congenital, originating from a variety of vessel wall dysfunctions and subsequent repair processes [9].

The primary risk factors for the formation of pediatric aneurysms include pre-existing conditions such as infection, tumors, trauma, or dissection. Furthermore, it has been established that some genetic illnesses, such as Ehlers-Danlos syndrome, moyamoya disease, and Marfan syndrome, contribute to this condition [5]. IAs are uncommon in children, but when they do occur, the effects are typically more severe than in adults. Around two-thirds of cases had hemorrhage as the primary symptom, and the rate of rebleeding is higher than that of adult cases [4].

Historically, there has been a significant rate of traumatic instances involving infantile aneurysms; roughly 40% of pediatric aneurysms are the result of brain trauma from either accident or surgical treatments. In contrast, spontaneous dissecting aneurysms in the pediatric population are uncommon [8]. Several research studies have demonstrated that in pediatric patients, cerebral aneurysms are frequently located distant from the Willis Circle. Given their rarity and potential for severe consequences, aggressive therapy is recommended. Surprisingly, patients with poor clinical grades at presentation can have outstanding results. Long-term surveillance imaging is necessary because of the possibility of aneurysm recurrence [10].

Adult individuals who have had an SAH are known to be at high risk for adverse outcomes due to cerebral vasospasm. According to a comprehensive analysis, adults treated with oral calcium antagonists may have a lower chance of unfavorable neurological outcomes. However, the evidence regarding the implications and management of vasospasm in children, especially infants, is limited. Some studies in pediatric populations have not shown a clear correlation between vasospasm and poor outcomes, leading to a lack of clear recommendations for the management of vasospasm in infants [11]. Antibiotics constitute the cornerstone of treatment for unruptured mycotic aneurysms. The literature suggests a recommended duration of four to six weeks of antibiotic therapy, paired with regular CT scans and angiography, to evaluate the state of the mycotic aneurysm [12].

In individuals with infective endocarditis, the reported incidence of mycotic cerebral aneurysms is between 2% and 3%. This number, though, might be overestimated because many people experience no symptoms, and the aneurysm might heal after receiving antibiotic treatment. The “vasa vasorum theory,” which postulates that microorganisms from embolic vegetation escape through the vasa vasorum and cause significant adventitial inflammation and damage, best explains the etiology. Associated with highly high fatality rates, mycotic aneurysms are uncommon but severe consequences of infective endocarditis. Significant complications arise in the management of infective endocarditis compounded by a mycotic aneurysm [13]. However, our case is not associated with infective endocarditis. A 2D echocardiography and blood culture were not suggestive of infective endocarditis.

Anatomically, the MCA is divided into four segments: sphenoidal (M1), opercular (M3), insular (M2), and cortical (M4). MCA bifurcation aneurysms account for the majority (80-96%) of all MCA aneurysms, making them the most prevalent type. Distal MCA segments (M3, M4) are home to sporadic aneurysms, accounting for around 6%-20% of all MCA aneurysms. Even less common are cortical (M4) MCA aneurysms, whose existence usually suggests the existence of an underlying pathogenic condition such as an infection, inflammation (vasculitis), trauma, or idiopathic etiology. Exceedingly uncommon is the idiopathic subtype of cortical MCA aneurysms [14].

The neurovascular intervention aims to protect cerebral vasculature, reduce the mass effect, and minimize the danger of aneurysmal hemorrhage. Cerebral aneurysms can be treated by endovascular coiling or surgical clipping. When choosing the best course of action, essential factors include the patient's clinical presentation, the aneurysm's size, morphology, etiology, location, and the parent vessel's characteristics [15]. Endovascular coiling or surgical clipping may be preferred depending on the patient's age and symptoms. Endovascular coiling has a lower death rate and requires less time in the hospital than clip placement. Nevertheless, there needs to be more thorough outcome data on these treatments in pediatric instances. Options for microsurgical procedures include bypass with proximal occlusion, trapping, and clip repair. Remarkably, obliterating the aneurysm sac surgically has been linked to lower rates of recurrence and, consequently, a decrease in the formation of new aneurysms [16].

A MCA aneurysm was clipped in this case after a craniotomy was performed. After the operation, the patient's activity improved, and his left upper and lower limb weakness gradually decreased. His left-sided movements also gradually improved. Nevertheless, the patient's facial deformity remained until their release. For the best results, it is essential to identify the condition as soon as possible, diagnose it, and treat it with a multidisciplinary team that includes specialists in neurology, neurosurgery, and rehabilitation. This example emphasizes the need for careful surgical monitoring and follow-up in pediatric patients with IAs and the possibility of transient neurological sequelae. More investigation and case studies are necessary to better understand the etiology, best practices for diagnosis, and treatment options for this uncommon illness in babies.

## Conclusions

This case report highlights the rarity and complexity of managing IAs in infants, as seen in our nine-month-old patient who presented with convulsions and SAH. Prompt diagnosis through advanced imaging led to successful surgical intervention via right craniotomy and aneurysm clipping, resulting in gradual improvement in limb movements and overall stability, although some neurological sequelae persisted. This case emphasizes the critical role of a multidisciplinary approach involving neurology, neurosurgery, and rehabilitation for optimal outcomes. It also underscores the need for ongoing research and case studies to better understand, diagnose, and treat this uncommon condition in pediatric patients.
